# Fetal Hypoxia Impacts on Proliferation and Differentiation of Sca-1^+^ Cardiac Progenitor Cells and Maturation of Cardiomyocytes: A Role of MicroRNA-210

**DOI:** 10.3390/genes11030328

**Published:** 2020-03-20

**Authors:** Xianmei Meng, Peng Zhang, Lubo Zhang

**Affiliations:** Lawrence D. Longo, MD Center for Perinatal Biology, Department of Basic Sciences, Loma Linda University School of Medicine, Loma Linda, CA 92350, USA; xmeng@llu.edu (X.M.); pzhang@llu.edu (P.Z.)

**Keywords:** hypoxia, microRNA-210, cardiac progenitor cells, congenital heart defects, differentiation, maturation

## Abstract

Hypoxia is one of the most frequent and severe stresses to an organism’s homeostatic mechanisms, and hypoxia during gestation has profound adverse effects on the heart development increasing the occurrence of congenital heart defects (CHDs). Cardiac progenitor cells (CPCs) are responsible for early heart development and the later occurrence of heart disease. However, the mechanism of how hypoxic stress affects CPC fate decisions and contributes to CHDs remains a topic of debate. Here we examined the effect of hypoxic stress on the regulations of CPC fate decisions and the potential mechanism. We found that experimental induction of hypoxic responses compromised CPC function by regulating CPC proliferation and differentiation and restraining cardiomyocyte maturation. In addition, echocardiography indicated that fetal hypoxia reduced interventricular septum thickness at diastole and the ejection time, but increased the heart rate, in mouse young adult offspring with a gender-related difference. Further study revealed that hypoxia upregulated microRNA-210 expression in Sca-1^+^ CPCs and impeded the cell differentiation. Blockage of microRNA-210 with LNA-anti-microRNA-210 significantly promoted differentiation of Sca-1^+^ CPCs into cardiomyocytes. Thus, the present findings provide clear evidence that hypoxia alters CPC fate decisions and reveal a novel mechanism of microRNA-210 in the hypoxic effect, raising the possibility of microRNA-210 as a potential therapeutic target for heart disease.

## 1. Introduction

Congenital heart defects (CHDs) represent the most common type of birth defects, with an incidence of 0.8% of live newborns in the United States (i.e., 35,000 annually) [1,2]. Although advances in surgical management have greatly improved survival, many children still experience reduced heart function and heart failure, which not only need frequent special care but also may require transplantation. CHDs affect more than one million adults in the United States alone [1,2]. Apart from a minority of CHD cases caused by genetic factors, the majority of CHD cases have been reported to be tightly linked with environmental factors including smoking, diabetes mellitus, obesity, and hypoxia [3,4,5,6]. Pregnancy at high altitude, with diseases of anemia, pulmonary or heart problems and preeclampsia, or placental insufficiency has been reported to cause hypoxic stress to the fetal development and increase the occurrence of CHDs [2,7,8]. These findings indicate that studies of understanding how hypoxic stress contributes to heart defects during cardiac morphogenesis are of clinical interest.

Cardiac stem/progenitor cells (CPCs) are tissue-specific stem cells that are identified based on the expression of certain transcription factors or cell surface markers. Isl1 and Nkx2.5 are two critical transcription factors, which are differentially expressed in different regions of fetal heart and strictly control cell lineage specification during cardiac development [9,10,11,12]. Stem cell antigen-1 (Sca-1) is one of useful cell surface markers, which has been selected to purify CPCs for clinical interest [13,14,15]. Intensive research has demonstrated that cardiac development follows a stem cell paradigm where a limited number of endogenous CPCs are responsible for generating all the major functional cell types including cardiomyocytes, endothelial cells and smooth muscle cells for cardiac myogenesis [16,17,18]. Any disruption in the step-wise processes of CPC commitment to differentiated progeny can cause cardiac malformation and CHDs [2]. Fate-mapping studies have shown that oxygen levels directly influence stem/progenitor cell-fate decisions [19,20], suggesting that hypoxic stress affects CPC fate and contributes to heart defects during cardiac morphogenesis. However, the mechanisms underlying hypoxic stress-induced effect on CPC fate decisions remain a topic of debate.

MicroRNAs are a family of non-coding RNAs (~22 nucleotides in length) that most commonly regulate gene expression by binding to complementary sites of the targeted mRNAs [21,22]. A substantial body of evidence has demonstrated that microRNAs participate in regulating CPC proliferation, differentiation and lineage specification by modulating cardiac gene expression [23,24,25,26]. MicroRNA-210 is regarded as a master microRNA of the hypoxic response due to its high popularity in all the tested cell types [27,28]. Our previous studies have shown that hypoxia promotes the ischemia-sensitive phenotype by increasing microRNA-210 expression during fetal heart development [5,29]. It is likely that microRNA-210 modulates CPC cell fate under hypoxia.

Here we examined the effect of hypoxic stress on the regulations of CPC fate decisions and the potential mechanism. We exposed time-dated pregnant CD-1 mice to 12% oxygen for 72 h from embryonic day 15 (E15) to embryonic day 18 (E18), and differentiated Sca-1^+^ CPCs to cardiomyocytes ex vivo under 1% oxygen. We found that experimental induction of hypoxic responses compromised CPC function by regulating CPC proliferation and differentiation and restraining cardiomyocyte maturation. In addition, echocardiography indicated that hypoxic stress reduced interventricular septum thickness at diastole (IVSd) and the ejection time, but increased the heart rate, in mouse young adult offspring with a gender-related difference. Further study revealed that hypoxia increased microRNA-210 expression in Sca-1^+^ CPCs and impeded the cell differentiation. Blockage of microRNA-210 with LNA-anti-microRNA-210 significantly promoted differentiation of Sca-1^+^ CPCs into cardiomyocytes. Our findings provide clear evidence that hypoxia alters CPC fate decisions and reveal a mechanism of microRNA-210 in the hypoxic effect, raising the possibility of microRNA-210 as a potential therapeutic target for heart disease.

## 2. Materials and Methods

### 2.1. Animal

Pregnant CD-1 mice were purchased from Charles River Laboratories (Wilmington, MA). Animals were allowed to give birth and were then kept with their pups in a room maintained at 20 ± 2 °C with a 12 h light–dark cycle. Time-dated pregnant CD-1 mice were randomly divided into two groups of normoxic control and hypoxic treatment. To induce hypoxic responses, the animals were placed in their home cages in a hypoxia chamber with 12% O_2_/88% N_2_ generated by an Altitude Generator (Everest Summit II, Hypoxico Altitude Training System, New York, NY, USA) from E15 to E18 for 72 h continuously. Oxygen in the chamber was measured using an oxygen analyzer (OxyCheq Expedition-X, FL, USA). The normoxic group was housed identically with room air flowing. Pups at day 21 were weaned and maintained till 4 weeks old at 20 ± 2 °C with a 12 h light–dark cycle. Animals were provided ad libitum access to normal mouse chow and water. All animal experiments were performed according to protocols approved by the Institutional Animal Care and Use Committee of Loma Linda University and followed the guidelines by the National Institutes of Health Guide for the Care and Use of Laboratory Animals.

### 2.2. Isolation of Primary Cardiac Cells and Sca-1^+^ Cardiac Progenitor Cells

Hearts from E15 and E19 embryos, postnatal day 7 (P7) and 14 (P14) pups, and 4-week old mice were used for isolating primary cardiac cells. Briefly, hearts were cut into small pieces and digested with 0.3 mg/mL Trypsin (Invitrogen, Carlsbad, CA, USA), 0.3 mg/mL collagenase II (Worthington, Lakewood, NJ, USA), 5.5 µg/mL DNase I (Sigma, St. Louis, MO) and 1 mg/mL BSA (Sigma) in HBSS solution (Gibco, Waltham, MA, USA) containing 1% penicillin-streptomycin (Gibco). Primary cardiac cells were obtained using Percoll (Sigma) gradient separation. For isolation of Sca-1^+^ CPCs, primary cardiac cells were first stained with CD31 beads (Miltenyi Biotec, Cat# 130-097-418) to deplete CD31^+^ cells using the Magnetic Cell Sorting System (MACS) (Miltenyi Biotec, San Diego, CA, USA), and then the negative portion was stained with Sca-1 beads (Miltenyi Biotec, Cat# 130-098-374) for MACS sorting.

### 2.3. In Vitro Cardiac Progenitor Cell Culture, Transfection, and Differentiation

Newly isolated Sca-1^+^ CPCs were cultured on 0.1% gelatin-coated well plates with the growth medium composed of EGM2 medium (Lonza, Allendale, NJ, USA) and M199 medium (Gibco) at the ratio of 1:3, supplemented with 10% FBS (Gibco), 20 ng/mL basic fibroblast growth factor (bFGF, Peprotech), 1% nonessential amino acids (Gibco), 100 µg/mL penicillin (Gibco), and 250 µg/mL of streptomycin (Gibco) plus 5 µM ROCK inhibitor Y-27632 (Sigma) at 37 °C in humid air with 5% CO_2_ overnight [15]. From the next day, the media was changed without ROCK inhibitor every 2–3 days. To measure the proliferation rates of Sca-1^+^ CPCs, cells were dissociated using 0.25% Trypsin (Gibco) for cell counting after culture under normoxia and hypoxia. Once the cell confluence reached ~85%, Sca-1^+^ CPCs were transfected with 50 nM LNA-anti-microRNA-210 (Exiqon, Germantown, MD, USA) or LNA scramble control (Exiqon) using optiMEM for 18 h [5]. Differentiation experiments were initiated with non-transfected and transfected Sca-1^+^ CPCs under normoxic (21% oxygen) and hypoxic (1% oxygen) conditions, respectively. Sca-1^+^ CPCs from three treatments of normoxia, hypoxia plus LNA-anti-microRNA-210, and hypoxia plus the scramble control were then treated with 5 µM of 5′-azacytizine in the differentiation medium composed of DMEM/F12 (Gibco) and IMDM (Gibco) at the ratio of 1:1, supplemented with 4% horse serum, 1% GlutaMAX (Gibco), 1% nonessential amino acids (Gibco), 1% insulin-transferrin-selenium (ITS, Gibco), 100 µg/mL penicillin (Gibco), and 250 µg/mL of streptomycin (Gibco) for 72 h, and freshly prepared 5′-azacytizine was added everyday [30]. Three days later, the differentiation medium was changed without 5’-azacytizine. From day 5, 20 µg/mL of ascorbic acid (Sigma) and 1 ng/mL TGF-β (Peprotech, Rocky Hill, NJ, USA) were added to the differentiation medium. Cells were maintained in this medium for 3~4 weeks with a medium change every 2~3 days. After the treatments, differentiated cells were harvested for staining by different antibodies for analysis.

### 2.4. Antibodies and Flow Cytometry

Fluorochrome-conjugated monoclonal antibodies specific for mouse Sca-1 (Cat#: 108125; Dilution: 1:200) was purchased from Biolegend (San Diego, CA, USA); and monoclonal antibodies specific for mouse MF20 (Cat#: 564408; Dilution: 1:100) and cTnT (Cat#: 564767; Dilution: 1:100) were purchased from BD Biosciences. Primary antibodies cTnT (Cat#: ab45932; Dilution: 1:200), Nkx2.5 (Cat#: PA5-49431; Dilution: 1:50) and Isl1 (Cat#: 40.2D6; Dilution: 1:25) were purchased from Abcam, ThermoFisher Scientific (Waltham, MA, USA) and Development Studies Hybriddoma Bank (DSHB), respectively. Secondary antibodies of Alexa Fluor 488 (Cat#: A32723; Dilution: 1:300) and Alexa Fluor 647 (Cat#: A27040; Dilution: 1:300) and Fixable Viability Dye eFluor® 506 (Cat#: 65-0866; Dilution: 1:1000) were purchased from ThermoFisher Scientific. Cells were stained with surface markers and then Fixable Viability Dye following a standard procedure. To detect intracellular proteins, cells were subjected to intracellular protein staining using a Cell Fixation/Permeabilization Kit (BD Biosciences) following the manufacturer’s instructions. Stained cells were analyzed by using a MACSQuant Analyzer 10 flow cytometer (Miltenyi Biotec). FlowJo software (Tree Star) was used to analyze the data.

### 2.5. MicroRNA Quantitative RT-PCR

Total RNA from cells was isolated using a miRNeasy Mini Kit (Qiagen, Germantown, MD, USA) and was reverse-transcribed with miScript II RT kit (Qiagen) following the manufacturer’s instructions. MicroRNA-210 expression was measured using miScript SYBR Green PCR kit with miScript Primer Assay kit (Qiagen) according to the manufacturer’s instructions. SNORD61 was used as an internal control. The relative expression of microRNA-210 was calculated by the 2^−ΔΔCT^ method and was presented as the fold induction relative to control. 

### 2.6. Echocardiography

Four-week old mice were subjected to transthoracic echocardiography using the LOGIQ E Ultrasound (GE Medical System, Chicago, IL, USA) with a 13 MHz probe (12L-RS). Briefly, one mouse each time was anaesthetized with 2% of isoflurane, the hair over the anterior chest was shaved and a layer of warm acoustic-coupling gel was applied over the thorax. The mouse was then placed in the left lateral decubitus position. The probe was positioned over the chest in a parasternal position. An M-mode recording of the left ventricular (LV) functions was obtained at the level of the mitral valve in the parasternal view using two-dimensional echocardiographic guidance in both the short and long axis views. Measurements and analysis were then performed using the methods described for mice [31] using AccessPoint software (Freeland Systems LLC, SantaFe, NM, USA).

### 2.7. Statistical Analysis

Paired comparisons were performed using the Student’s two-tailed *t* test. Multiple comparisons were performed using the ordinary one-way ANOVA followed by Tukey test. Data are presented as mean ± SEM, unless otherwise indicated. *p* ≤ 0.05 was considered significant (*, *p* ≤ 0.05; **, *p* ≤ 0.01; ***, *p* ≤ 0.001).

## 3. Results

### 3.1. Fetal Hypoxia Regulates CPC Proliferation and Restrains Cardiomyocyte Maturation in Mouse Fetal and Postnatal Hearts

**Figure 1:**
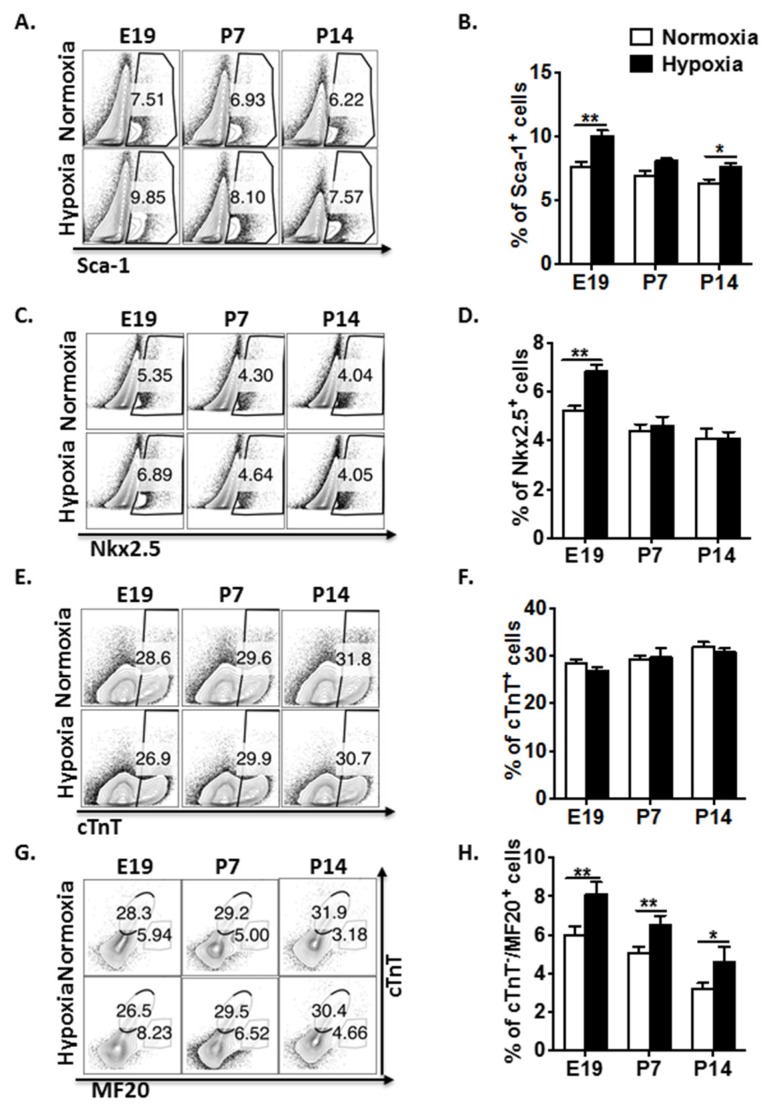
Hypoxia regulates cardiac progenitor cell (CPC) proliferation and restrains cardiomyocyte maturation in mouse fetal and postnatal hearts. (**A**) Representative flow plots showing the surface Sca-1 staining of cardiac cells after depletion of cardiomyocytes. (**B**) Quantification of the flow plots presented in (A). Data are presented as the mean ± SEM (n = 4). (**C**) Representative flow plots showing the intracellular Nkx2.5 staining of cardiac cells after depletion of cardiomyocytes. (**D**) Quantification of the flow plots presented in (C). Data are presented as the mean ± SEM (n = 4). (**E**) Representative flow plots showing the intracellular cTnT staining of cardiac cells. (**F**) Quantification of the flow plots presented in (E). Data are presented as the mean ± SEM (n = 4). (**G**) Representative flow plots showing the intracellular cTnT and MF20 staining of cardiac cells. (**H**) Quantification of the flow plots presented in (G). Data are presented as the mean ± SEM (n = 4). * *p* ≤ 0.05 and ** *p* ≤ 0.01.

In order to examine the effect of hypoxia on mouse heart development, we exposed time-dated pregnant CD-1 mice to low oxygen tension (12% oxygen) for 72 h from E15 to E18. At three time points of E19, P7, and P14, whole hearts from fetuses and pups were collected for isolating cardiac cells. Different populations of CPCs and cardiomyocytes were analyzed by flow cytometry. Sca-1^+^ cells and Nkx2.5^+^ cells accounted for approximately 6%~8% and 3%~7%, respectively, in control fetal and postnatal mouse hearts after excluding cardiomyocytes (Figure 1A–D), which is consistent with the previous studies [2,11,15,32]. We found that experimental induction of hypoxic responses significantly enhanced Sca-1 and Nkx2.5 expressions in cardiac cells at E19, compared to the normoxic control. This hypoxic stress-induced effect was sustained in the postnatal heart at P14 for Sca-1^+^, but not Nkx2.5^+^ cells. Isl1^+^ CPCs drop sharply in fetal heart from late embryonic stages and are very few in postnatal and adult hearts [32,33]. In the present study, Isl1^+^ cells were not detectable in fetal and postnatal hearts. cTnT expression follows the pattern of increasing expression with the age [34]. The similar trend of cTnT expression was noticed in our study, but the data did not show significant differences of cTnT expression in the fetal and postnatal hearts between normoxic and hypoxic groups (Figure 1E,F). In contrast, the immature cardiomyocytes (cTNT^−^/MF20^+^) decreased with the age, but hypoxia caused a significant and sustained increase in immature cardiomyocytes from fetal to postnatal P14 mouse hearts (Figure 1G,H). Collectively, these results reveal that hypoxic stress differentially regulates CPC proliferation and retards cardiomyocyte maturation in mouse fetal and postnatal hearts.

### 3.2. Antenatal Hypoxia Regulates CPC Proliferation and Restrains Cardiomyocyte Maturation with a Gender-Related Difference in Young Adult Mice

**Figure 2:**
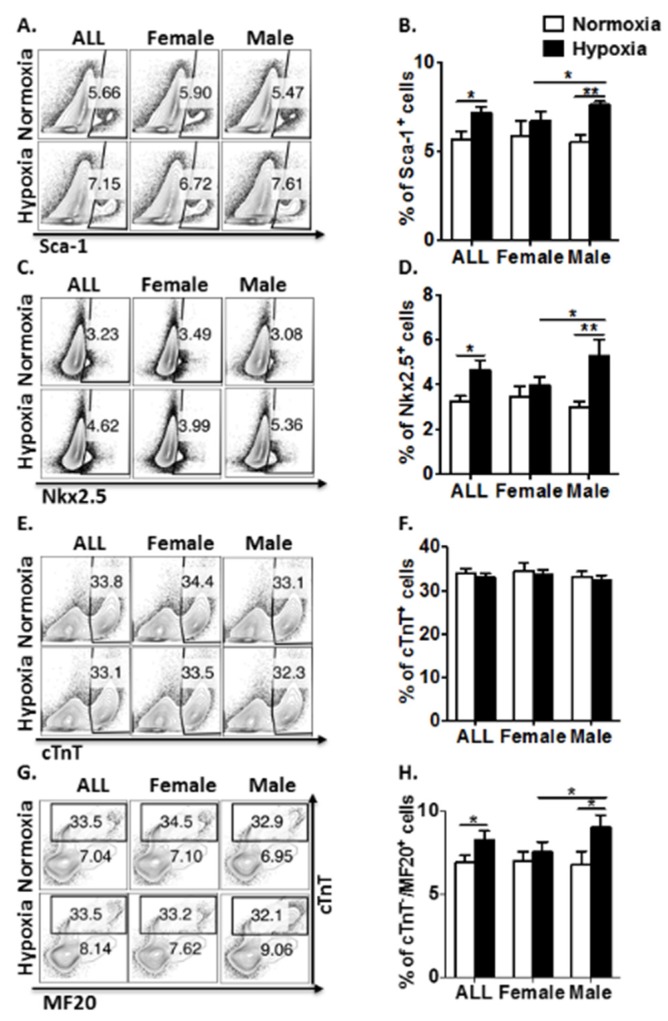
Prenatal hypoxia regulates CPC proliferation and restrains cardiomyocyte maturation in four-week-old offspring with a gender-related difference. (**A**) Representative flow plots showing the surface Sca-1 staining of cardiac cells after depletion of cardiomyocytes. (**B**) Quantification of the flow plots presented in (A). Data are presented as the mean ± SEM (n = 4). (**C**) Representative flow plots showing the intracellular Nkx2.5 staining of cardiac cells after depletion of cardiomyocytes. (**D**) Quantification of the flow plots presented in (C). Data are presented as the mean ± SEM (n = 4). (**E**) Representative flow plots showing the intracellular cTnT staining of cardiac cells. (**F**) Quantification of the flow plots presented in (E). Data are presented as the mean ± SEM (n = 4). (**G**) Representative flow plots showing the intracellular cTnT and MF20 staining of cardiac cells. (**H**) Quantification of the flow plots presented in (**G**). Data are presented as the mean ± SEM (n = 4). * *p* ≤ 0.05 and ** *p* ≤ 0.01.

To further investigate the long-term effect of antenatal hypoxic stress on the heart development in mouse young adult offspring, pups from normoxic and hypoxic groups were kept until four weeks old after birth. One day after transthoracic echocardiography, mouse hearts were collected for isolating cardiac cells. Analysis of cardiac cell population by flow cytometry showed that experimental induction of hypoxic responses resurged the significant effect on enhancing Sca-1 and Nkx2.5 expressions in cardiac cells after excluding cardiomyocytes (Figure 2A–D). In agreement with these results, the previous study demonstrated a resurgence of Nkx2.5 cell population during the first three weeks after birth [11]. Of interest, the effect of antenatal hypoxia is sex-dependent. As shown in Figure 2A–D, prenatal hypoxia did not cause a significant difference in Sca-1 and Nkx2.5 expressions in cardiac cells compared to the normal control in females, but significantly increased Sca-1^+^ and Nkx2.5^+^ cardiac cells in young adult males, compared to the normal control. Apparently, prenatal hypoxia significantly raised more Sca-1 and Nkx2.5 expressions in cardiac cells in males than those in females (Figure 2A–D). There was no significant difference of cTnT expression between normoxic control and hypoxic groups in both male and female cardiac cells (Figure 2E,F). Nevertheless, more immature cardiomyocytes were observed in male mouse hearts from hypoxic group than those from normoxic group (Figure 2G,H). Likewise, prenatal hypoxia did not cause significant difference in the number of immature cardiomyocytes compared to the normal control in females. Instead, prenatal hypoxia significantly increased the number of immature cardiomyocytes, compared to the normal control in males (Figure 2G,H). Moreover, prenatal hypoxia significantly raised more immature cardiomyocytes in males than those in females (Figure 2G,H). Taken together, these results document that regulation of CPC proliferation and restraint of cardiomyocyte maturation by prenatal hypoxia shows a gender-related difference in young adult offspring.

### 3.3. Prenatal Hypoxia Impairs Heart Function with a Gender-Related Difference in Young Adult Mice

**Figure 3:**
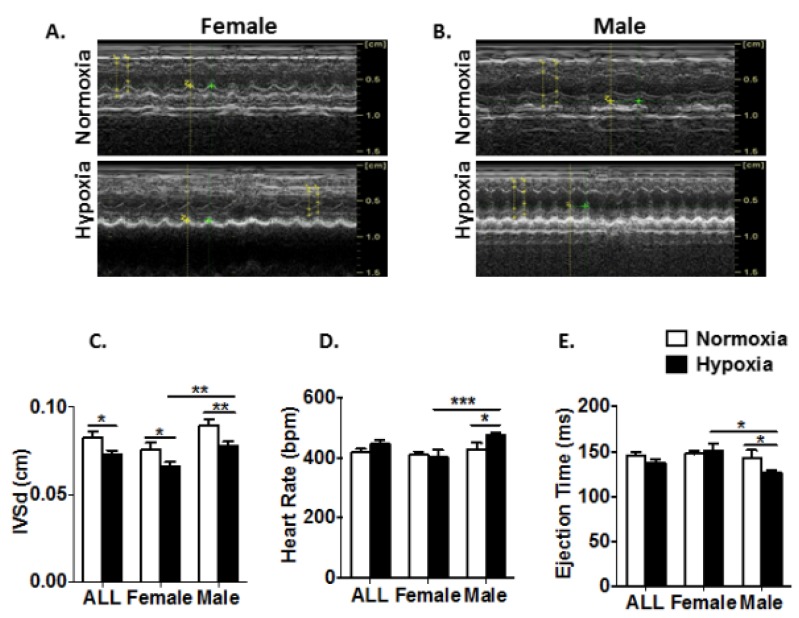
Prenatal hypoxia impairs heart function in four-week-old offspring with a gender-related difference. Mice were studied by echocardiography at four weeks old using an M-mode recording of the left ventricular (LV) at the level of the mitral valve in the parasternal view. (**A**) Representative echocardiographic images of M-model measurement of LV structures for females under the indicated conditions. (**B**) Representative echocardiographic images of M-model measurement of LV structures for males under the indicated conditions. (**C**) Interventricular septum thickness at diastole (IVSd) for all mice under the indicated conditions. Data are presented as the mean ± SEM (n_ALL-Normoxia_ = 8 and n_ALL-Hypoxia_ = 12; n_Female-Normoxia_ = 4 and n_Female-Hypoxia_ = 5; n_Male-Normoxia_ = 4 and n_Female-Hypoxia_ = 7). (**D**) Ejection time for all mice under the indicated conditions. Data are presented as the mean ± SEM (n_ALL-Normoxia_ = 8 and n_ALL-Hypoxia_ = 12; n_Female-Normoxia_ = 4 and n_Female-Hypoxia_ = 5; n_Male-Normoxia_ = 4 and n_Female-Hypoxia_ =7). (E) Heart rate for all mice under the indicated conditions. Data are presented as the mean ± SEM (n_ALL-Normoxia_ = 8 and n_ALL-Hypoxia_ = 12; n_Female-Normoxia_ = 4 and n_Female-Hypoxia_ = 5; n_Male-Normoxia_ = 4 and n_Female-Hypoxia_ =7). * *p* ≤ 0.05, ** *p* ≤ 0.01 and *** *p* ≤ 0.001.

To evaluate the effect of prenatal hypoxia on the heart function in mouse young adult offspring, an M-mode echocardiography was used to record the left ventricle (LV) structure and function at the level of the mitral valve in the parasternal view in four-week-old offspring after birth. Representative images showed echocardiographic measurements of LV structures for females (Figure 3A) and males (Figure 3B), respectively, under the indicated conditions. In comparison with normoxic control offspring, offspring treated with prenatal hypoxia showed on echocardiography a reduction in interventricular septum thickness at diastole (IVSd) in all three comparisons (Figure 3C). Prenatal hypoxia did not affect the heart rate compared to the normoxic control in females. Interestingly, prenatal hypoxia resulted in a significant increase in the heart rate in males, compared to the normoxic control (Figure 3D). In addition, prenatal hypoxia did not affect the ejection time compared to the normal control in females. However, it significantly decreased the ejection time compared to the normal control in males (Figure 3D). Thus, echocardiographic measurements of LV illustrate that young males were more susceptible to prenatal hypoxia than females, revealing that antenatal hypoxia impairs the heart function of mouse young adult offspring with a gender-related difference (Figure 3C,D).

### 3.4. Hypoxia Induces MicroRNA-210 Expression in Sca-1^+^ CPCs and Inhibits Their Differentiation to Cardiomyocytes Ex Vivo

**Figure 4:**
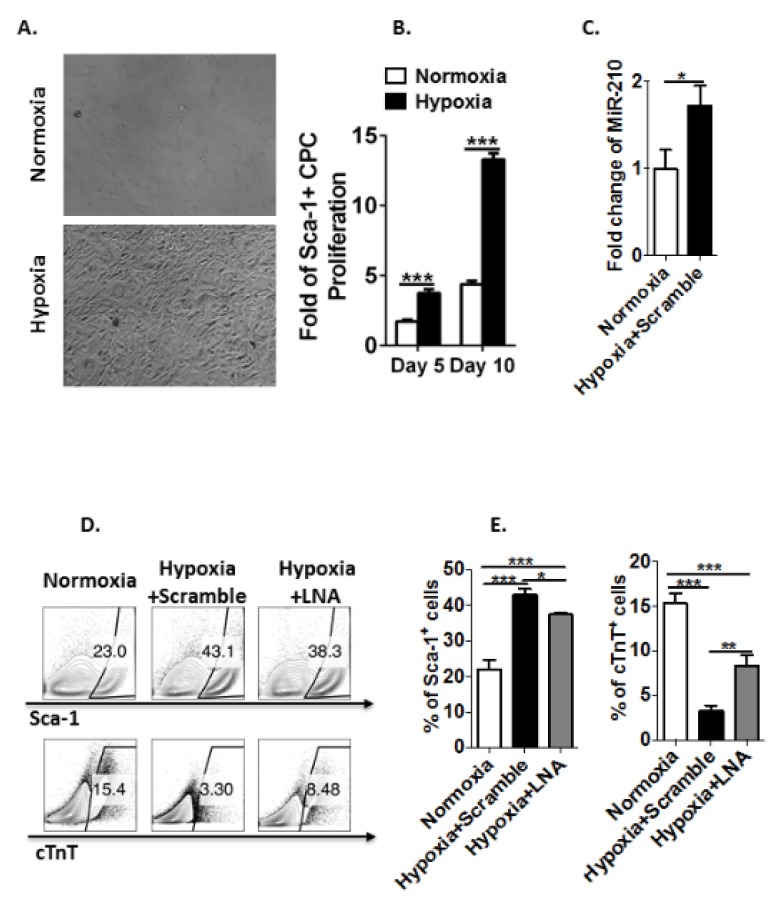
Hypoxia induces microRNA-210 expression in Sca-1^+^ CPCs and inhibits their differentiation to cardiomyocytes ex vivo. (**A**) Bright-field images of Sca-1^+^ CPCs cultured under normoxia and hypoxia. (**B**) Fold change of Sca-1^+^ CPC proliferation under normoxia and hypoxia. Data are presented as the mean ± SEM (n = 3). (**C**) Quantitative RT-PCR analysis of microRNA-210 expression in Sca-1^+^ CPCs cultured under the indicated conditions. Data are presented as the mean ± SEM (n = 3). (**D**) Representative flow plots showing the surface Sca-1 staining and intracellular cTnT staining of Sca-1^+^ CPCs after differentiation into cardiomyocytes. (**E**) Quantification of the flow plots presented in (D). Data are presented as the mean ± SEM (n = 3). * *p* ≤ 0.05, ** *p* ≤ 0.01 and *** *p* ≤ 0.001. LNA: LNA-anti-miR-210.

To access the possible mechanism of regulation of CPC proliferation and restraint of cardiomyocyte maturation by hypoxia, cardiac cells were isolated from whole hearts of E15 fetal mice and Sca-1+ CPCs were sorted by MACS beads for the ex vivo study. Newly-sorted Sca-1^+^ CPCs were cultured for expansion under normoxic (21% oxygen) and hypoxic (1% oxygen) conditions, respectively. We observed that Sca-1^+^ CPCs expanded ~1.7-fold by day 5 and ~4.4-fold by day 10 under normoxic culture, while they expanded ~4-fold by day 5 and ~13-fold by day 10 under hypoxic culture (Figure 4A,B). Quantitative RT-PCR analysis of microRNA-210 expression in Sca-1^+^ CPCs harvested from normoxic and hypoxic conditions revealed an upregulation of microRNA-210 expression in these cells cultured under the hypoxic condition (Figure 4C). To directly assay the differentiation capacity of Sca-1^+^ CPCs into cardiomyocytes under hypoxia, newly-sorted Sca-1^+^ CPCs were first cultured under normoxia to reach a confluence of ~85%. Sca-1^+^ CPCs were then transfected with LNA-anti-microRNA-210 (Hypoxia plus LNA) or its scramble control (Hypoxia plus Scramble) or without transfection (Normoxia). About three to four weeks after differentiation experiment, analysis of differentiated cells showed that hypoxia dramatically maintained a higher percentage of Sca-1^+^ CPCs in the treatments of hypoxia plus scramble control (~43%) than that in the treatment of normoxia (~23%) (Figure 4D,E). At the same time, hypoxia dramatically impeded differentiation of Sca-1^+^ CPCs into cardiomyocytes with a differentiation percentage of ~3% in the treatment of hypoxia plus scramble control versus ~15% in that of normoxia (Figure 4D,E). Of importance, blockage of microRNA-210 with LNA-anti-microRNA-210 significantly inhibited the effects of hypoxia and resumed the differentiation capacity of Sca-1^+^ CPCs under hypoxia with a differentiation percentage of ~8% in the treatment of hypoxia plus LNA versus ~3% in that of hypoxia plus scramble control (Figure 4D,E). Hence, the results suggest that inhibition of microRNA-210 reverses the effect of hypoxic stress on differentiation of Sca-1^+^ CPCs to cardiomyocytes.

## 4. Discussion

The compelling evidence of the present study demonstrates that fetal hypoxia regulates CPC proliferation and differentiation as well as restrains cardiomyocyte maturation to impair heart development and function after birth with a gender-related difference. To buttress this argument, analysis of cardiac cell population reveals that prenatal hypoxia causes more adverse effects on male offspring than on female ones. In addition, echocardiography indicates that prenatal hypoxia leads to a stronger reduction of IVSd and the ejection time and a greater increased heart rate in male offspring than in female ones. Thus, prenatal hypoxia impedes heart development and impairs heart function with a gender-related difference in mouse young adult offspring. Consistency with the previous studies was that prenatal exposure to hypoxia impairs heart function in later adult life with a gender-related difference [35,36,37]. Moreover, the present study has disclosed that hypoxia upregulates microRNA-210 expression in Sca-1^+^ CPCs and blockage of microRNA-210 with LNA-anti-microRNA-210 significantly promoted differentiation of Sca-1+ CPCs into cardiomyocytes.

Cardiac development adheres to a stem cell paradigm and endogenous CPCs can generate all the major functional cells for cardiac myogenesis [14,16,17,18,32]. Previous studies have reported that oxygen levels play a critical role in stem/progenitor cell-fate decisions and cardiac development [19,20,38,39,40]. Non-physiological hypoxia, as a major challenge to fetuses during the gestation due to the reduction in oxygen delivery to the developing fetus, has been reported to increase the occurrence of congenital cardiac anomalies [8,35,39,41]. Thus, the role of hypoxia in regulation of CPC proliferation and differentiation for cardiac malformations has gained increasing attentions. Several studies have shown that hypoxic stress destroys the balance between the amounts of oxygen supplied to the cardiac cells and of that needed, which disrupts the step-wise processes of CPC commitment to differentiated progeny [2,42]. Severe hypoxia compromises CPC function via down-regulating c-Myc protein stability [38]. In addition, hypoxia directly or indirectly alters the expression of many genes that are involved in stem cell proliferation and differentiation [40,42]. Hypoxia ceases Isl1 expression and activates Nkx2.5 expression by recruitment of the protein deacetylase sirtuin 1 to break the homeostasis of CPCs [2]. Low oxygen levels have been reported to maintain self-renew and an undifferentiated state of stem cells and improve their proliferative capacity by activating canonical Wnt pathway signaling and PI3K/Akt pathway [43]. Prenatal hypoxia leads to an increase in cardiac vulnerability to cardiovascular dysfunction in later life through upregulation of Akt pathway [41,44]. A recent study has shown that short-term preconditioning of CPCs by hypoxia improves CPC proliferation and survival to enhance the cell function ex vivo [45]. Cardiomyocytes occupy around one third of the cells found in the heart. cTnT is a cardiomyocyte specific marker and does not express in CPCs. In our study, the high basal levels of cTnT expression masked the small percentage changes of cardiomyocytes differentiated from endogenous CPCs caused by hypoxia (Figure 1E,F; Figure 2E,F). In our in vitro study, we directly differentiated isolated Sca-1^+^ CPCs into cardiomyocytes. Thus, compared to the differences of cTnT expression change in vivo, the difference of CPC differentiation into cardiomyocytes in vitro between under hypoxia and under normoxia was much more significant (Figure 4D,E). Our study demonstrates that hypoxia not only regulates CPC proliferation and differentiation, but also restrains cardiomyocyte maturation to impair heart development and function. Overall, these findings indicate that hypoxic stress affects CPC fate and contributes to heart defects during cardiac morphogenesis.

It is generally accepted that insults at critical stages of heart development may lead to permanent changes in tissue structure and function. As the first organ to form in the embryo, hearts fully develop during the later stages and function at birth. Exposure to 12% of hypoxic stress from E15 to E21 impairs rat offspring’s vascular function in later life [41]. Hypoxic insult by 8% oxygen for 24 h causes a lethal rate of 89% of E13 fetuses, but 5% of E11.5 and 51% of E17.5 fetuses [46], indicating E13 is a very sensitive heart development stage. Using CD-1 mice, this work further proves the detachment of epicardium and the thinning of myocardium by hypoxic stress [46]. In our initial experiments, E13 pregnant CD-1 mice were treated with 10% oxygen for three days, and we found that most fetuses were absorbed or died after birth. These findings validate that maternal hypoxia can cause growth restriction and even lethality. It has been shown that hearts from male offspring appear more vulnerable to hypoxic stress than those from female ones [35,36,37,47]. In addition, Zhao et al. demonstrated that hypoxia induced the activation of signal pathway controlling cell fate with a gender-related difference in cardiac fibroblasts [48]. Female cells are relatively resistant to hypoxia-induced inhibition in DNA synthesis, but male cells are susceptible [48]. In the present study, we performed a hypoxia of 12% oxygen on pregnant CD-1 mice from E15 to E18. The selected oxygen level is equivalent to over 4500 m above sea level as calculated by altitude air pressure calculator. At this altitude, the standard barometric pressure is similar to the situation seen in pregnant mothers exposed to extremely high altitude. Echocardiography was used to examine the heart functions in our study, so we chose to check four-week-old offspring after hypoxic treatment. Our previous study has reported that prenatal hypoxia decreased sheep fetal heart weights in a sex-related manner [29], indicating that the sex-dependent response to hypoxia might have already manifested at an earlier stage than four weeks old in mice. We observed significant increases in Sca-1^+^ and Nkx2.5^+^ cardiac cells as well as immature cardiomyocytes in young adult males in a sex-dependent manner, resulting from antenatal hypoxia. Consistently, echocardiography showed a reduction of IVSd and the ejection time and an increase in the heart rate in a sex-dependent manner in male offspring that exposed to fetal hypoxia. The thickness of IVSd is associated with heart physiology and functions. We did not detect significant changes in heart rate and ejection time at four weeks old in female offspring, which suggests that it may require a longer time follow-up in female. Primary Sca-1^+^ CPCs were isolated from E15 mouse hearts for the in vitro study (Figure 4). We aimed to use Sca-1^+^ CPCs to explore the possible mechanisms on how hypoxia impeded CPC differentiation to cardiomyocytes. Thus, we did not compare the differences between primary cells from E15 female and male fetuses. Further investigation of whether sex differences influence CPC differentiation potential under hypoxia is warranted. Therefore, accumulating evidence suggests that antenatal hypoxia contributes to heart defects during cardiac morphogenesis impacting cardiac structure and function in offspring with a gender-related difference. 

MicroRNAs have been tightly associated to the initiation and development of heart disease due to their role of “fine-tuning” cardiac gene expression to regulate the CPC fate. In particular, several microRNAs have been reported to play important roles in regulating CPC proliferation and differentiation as well as cardiomyocyte proliferation [21,49]. MicroRNA-21 efficiently accelerates proliferation of c-kit+ CPCs through targeting PTEN/PI3K/Akt signaling [26]. Similarly, microRNA-21 promotes proliferation of cultured rat neural stem/progenitor cells after hypoxic stimulation by activating Akt signaling pathway [22]. MicroRNA-218 promotes proliferation and inhibits differentiation in mouse CPCs through canonical Wnt signaling pathway [25]. On the other hand, microRNA-21 increases in CPC-derived exosomes under oxidative stress to protect cardiomyocytes from apoptosis through downregulating programmed cell death 4 (PDCD4) [23]. MicroRNA-133a has been identified as critical components of a myogenic transcriptional circuit to regulate cardiomyocyte proliferation and inhibit smooth muscle gene expression [49]. MicroRNA-210 modulates differentiation and migration of endothelial cells under hypoxia via downregulating Ephrin-A3 expression [50]. To explore the mechanisms on how hypoxia impeded differentiation of Sca-1^+^ CPCs to mature cardiomyocytes, we isolated Sca-1^+^ CPCs and directly differentiated them into cardiomyocytes. Sca-1 as the most representative marker for this subpopulation was used to measure the differentiation capabilities under the indicated conditions. Markers Nkx2.5 and Isl1 for other subpopulations were not analyzed. Compared to MF20, cTnT is a specific marker for mature cardiomyocytes that exhibit structural maturity and execute the functions in the heart. Therefore, analysis of Sca-1 for CPC subpopulation and cTnT for mature cardiomyocytes is sufficient for the in vitro study (Figure 4D,E). Our previous studies have shown that hypoxia-induced microRNA-210 expression increases fetal heart susceptibility to ischemia injury [5,29]. We have characterized the microRNA-210 promoter and identified the hypoxia response element HRE-63 as the HIF-1α binding site responsible for the robust induction of the microRNA-210 promoter in response to hypoxia in cardiomyocytes [5]. Similar findings in mouse and human showed that microRNA-210 promoter harbors three HREs and the HRE3 is identified as the HIF-1α binding site responsible for the robust induction of microRNA-210 promoter activity [51,52]. We and others have shown that hypoxia increases microRNA-210 expression in both fetal and neonatal cardiomyocytes [5,53]. Glucocorticoid receptor (GR) signaling pathways play a critical role in proper cardiomyocyte development and over all cardiac function. Our previous study demonstrated that the GR is a downstream target of microRNA-210 and HIF-1α-dependent microRNA-210-mediated GR suppression increased cardiomyocyte apoptosis in rat primary fetal heart [5]. Blockage of hypoxia-induced reduction of GR protein by LNA-anti-microRNA-210 alleviated cardiomyocyte apoptosis [5]. A recent study has shown that hypoxia-induced microRNA-210 promoted apoptosis of mouse spermatocyte GC-2 cells by directly targeting Kruppel-like factor 7 [54]. Our present study demonstrated that hypoxia significantly upregulated microRNA-210 expression in Sca-1^+^ cardiac cells and increased Sca-1+ cell proliferation. Of importance, inhibition of endogenous microRNA-210 with microRNA-210-LNA significantly decreased hypoxia-induced Sca-1+ cell proliferation, providing novel evidence of a causal role of microRNA-210 in hypoxia-mediated CPC proliferation. In addition, the inhibition of microRNA-210 significantly rescued differentiation capacity of Sca-1^+^ CPCs into cTnT^+^ cardiomyocytes under hypoxia, indicating the great potential of a functional restoration. Currently, no evidence could support that miR-210 directly targets cardiomyocyte markers of differentiation or that LNA-anti-miR-210 can directly restore myocardial function. However, these finding suggest a vital role of microRNA-210 in regulating hypoxia-mediated CPC fate and cardiomyocyte maturation in the developing heart. Given that mitochondrial function plays a critical role in the regulation of stem cell proliferation and differentiation and it is a major downstream target of microRNA-210, further investigation is needed to explore the mechanisms of mitochondria in microRNA-210 modulating CPC fate and cardiomyocyte maturation under hypoxia.

In a clinical study, microRNA-210 has been identified as a biomarker for CHDs [55]. The present study highlights that inhibition of microRNA-210 significantly resumes the differentiation capacity of CPCs and promotes cardiomyocyte maturation under hypoxia. Thus, the present finding raises the possibility of microRNA-210 as a promising therapeutic target for heart disease.

## References

[1] Bardot E., Calderon D., Santoriello F., Han S., Cheung K., Jadhav B., Burtscher I., Artap S., Jain R., Epstein J. (2017). Foxa2 identifies a cardiac progenitor population with ventricular differentiation potential. Nat. Commun..

[2] Yuan X., Qi H., Li X., Wu F., Fang J., Bober E., Dobreva G., Zhou Y., Braun T. (2017). Disruption of spatiotemporal hypoxic signaling causes congenital heart disease in mice. J. Clin. Investig..

[3] Stothard K.J., Tennant P.W., Bell R., Rankin J. (2009). Maternal overweight and obesity and the risk of congenital anomalies: A systematic review and meta-analysis. JAMA.

[4] Correa A., Gilboa S.M., Besser L.M., Botto L.D., Moore C.A., Hobbs C.A., Cleves M.A., Riehle-Colarusso T.J., Waller D.K., Reece E.A. (2008). Diabetes mellitus and birth defects. Am. J. Obstet. Gynecol..

[5] Martinez S.R., Ma Q., Dasgupta C., Meng X., Zhang L. (2017). MicroRNA-210 suppresses glucocorticoid receptor expression in response to hypoxia in fetal rat cardiomyocytes. Oncotarget.

[6] Xiao D., Wang L., Huang X., Li Y., Dasgupta C., Zhang L. (2016). Protective Effect of Antenatal Antioxidant on Nicotine-Induced Heart Ischemia-Sensitive Phenotype in Rat Offspring. PLoS ONE.

[7] Stout K. (2005). Pregnancy in women with congenital heart disease: The importance of evaluation and counselling. Heart.

[8] Sun L., Macgowan C.K., Sled J.G., Yoo S.J., Manlhiot C., Porayette P., Grosse-Wortmann L., Jaeggi E., McCrindle B.W., Kingdom J. (2015). Reduced fetal cerebral oxygen consumption is associated with smaller brain size in fetuses with congenital heart disease. Circulation.

[9] Sun Y., Liang X., Najafi N., Cass M., Lin L., Cai C.L., Chen J., Evans S.M. (2007). Islet 1 is expressed in distinct cardiovascular lineages, including pacemaker and coronary vascular cells. Dev. Biol..

[10] Lien C.L., Wu C., Mercer B., Webb R., Richardson J.A., Olson E.N. (1999). Control of early cardiac-specific transcription of Nkx2-5 by a GATA-dependent enhancer. Development.

[11] Serpooshan V., Liu Y.H., Buikema J.W., Galdos F.X., Chirikian O., Paige S., Paige S., Venkatraman S., Kumar A., Rawnsley D.R. (2017). Nkx2.5+ Cardiomyoblasts Contribute to Cardiomyogenesis in the Neonatal Heart. Sci. Rep..

[12] Moretti A., Caron L., Nakano A., Lam J.T., Bernshausen A., Chen Y., Qyang Y., Bu L., Sasaki M., Martin-Puig S. (2006). Multipotent embryonic isl1+ progenitor cells lead to cardiac, smooth muscle, and endothelial cell diversification. Cell.

[13] Pfister O., Mouquet F., Jain M., Summer R., Helmes M., Fine A., Colucci W.S., Liao R. (2005). CD31- but Not CD31+ cardiac side population cells exhibit functional cardiomyogenic differentiation. Circ. Res..

[14] Takamiya M., Haider K.H., Ashraf M. (2011). Identification and characterization of a novel multipotent sub-population of Sca-1(+) cardiac progenitor cells for myocardial regeneration. PLoS ONE.

[15] Wang H., Chen H., Feng B., Wang X., He X., Hu R., Yin M., Wang W., Fu W., Xu Z. (2014). Isolation and characterization of a Sca-1+/CD31- progenitor cell lineage derived from mouse heart tissue. BMC Biotechnol..

[16] Banerjee I., Fuseler J.W., Price R.L., Borg T.K., Baudino T.A. (2007). Determination of cell types and numbers during cardiac development in the neonatal and adult rat and mouse. Am. J. Physiol. Heart Circ. Physiol..

[17] Lescroart F., Wang X., Lin X., Swedlund B., Gargouri S., Sanchez-Danes A., Moignard V., Dubois C., Paulissen C., Kinston S. (2018). Defining the earliest step of cardiovascular lineage segregation by single-cell RNA-seq. Science.

[18] Santini M.P., Forte E., Harvey R.P., Kovacic J.C. (2016). Developmental origin and lineage plasticity of endogenous cardiac stem cells. Development.

[19] Kusuma S., Peijnenburg E., Patel P., Gerecht S. (2014). Low oxygen tension enhances endothelial fate of human pluripotent stem cells. Arterioscler. Thromb. Vasc. Biol..

[20] Kimura W., Xiao F., Canseco D.C., Muralidhar S., Thet S., Zhang H.M., Abderrahman Y., Chen R., Garcia J.A., Shelton J.M. (2015). Hypoxia fate mapping identifies cycling cardiomyocytes in the adult heart. Nature.

[21] Li B., Meng X., Zhang L. (2018). microRNAs and cardiac stem cells in heart development and disease. Drug Discov. Today.

[22] Chen R., Liu Y., Su Q., Yang Y., Wang L., Ma S., Yan J., Xue F., Wang J. (2017). Hypoxia stimulates proliferation of rat neural stem/progenitor cells by regulating mir-21, an in vitro study. Neurosci. Lett..

[23] Xiao J., Pan Y., Li X.H., Yang X.Y., Feng Y.L., Tan H.H., Jiang L., Feng J., Yu X.Y. (2016). Cardiac progenitor cell-derived exosomes prevent cardiomyocytes apoptosis through exosomal miR-21 by targeting PDCD4. Cell Death Dis..

[24] Deng S., Zhao Q., Zhou X., Zhang L., Bao L., Zhen L., Zhang Y., Fan H., Liu Z., Yu Z. (2016). Neonatal Heart-Enriched miR-708 Promotes Differentiation of Cardiac Progenitor Cells in Rats. Int. J. Mol. Sci..

[25] Wang Y., Liu J., Cui J., Sun M., Du W., Chen T., Ming X., Zhang L., Tian J., Li J. (2016). MiR218 Modulates Wnt Signaling in Mouse Cardiac Stem Cells by Promoting Proliferation and Inhibiting Differentiation through a Positive Feedback Loop. Sci. Rep..

[26] Shi B., Deng W., Long X., Zhao R., Wang Y., Chen W., Xu G., Sheng J., Wang D., Cao S. (2017). miR-21 increases c-kit(+) cardiac stem cell proliferation in vitro through PTEN/PI3K/Akt signaling. PeerJ.

[27] Kulshreshtha R., Ferracin M., Wojcik S.E., Garzon R., Alder H., Agosto-Perez F.J., Davuluri R., Liu C.G., Croce C.M., Negrini M. (2007). A microRNA signature of hypoxia. Mol. Cell. Biol..

[28] Huang X., Le Q.T., Giaccia A.J. (2010). MiR-210--micromanager of the hypoxia pathway. Trends Mol. Med..

[29] Zhang P., Ke J., Li Y., Huang L., Chen Z., Huang X., Zhang L., Xiao D. (2019). Long-term exposure to high altitude hypoxia during pregnancy increases fetal heart susceptibility to ischemia/reperfusion injury and cardiac dysfunction. Int. J. Cardiol..

[30] Smits A.M., van Vliet P., Metz C.H., Korfage T., Sluijter J.P., Doevendans P.A., Goumans M.J. (2009). Human cardiomyocyte progenitor cells differentiate into functional mature cardiomyocytes: An in vitro model for studying human cardiac physiology and pathophysiology. Nat. Protoc..

[31] Nakada Y., Canseco D.C., Thet S., Abdisalaam S., Asaithamby A., Santos C.X., Shah A.M., Zhang H., Faber J.E., Kinter M.T. (2017). Hypoxia induces heart regeneration in adult mice. Nature.

[32] Laugwitz K.L., Moretti A., Lam J., Gruber P., Chen Y., Woodard S., Lin L.Z., Cai C.L., Lu M.M., Reth M. (2005). Postnatal isl1+ cardioblasts enter fully differentiated cardiomyocyte lineages. Nature.

[33] Khattar P., Friedrich F.W., Bonne G., Carrier L., Eschenhagen T., Evans S.M., Schwartz K., Fiszman M.Y., Vilquin J.T. (2011). Distinction between two populations of islet-1-positive cells in hearts of different murine strains. Stem Cells Dev..

[34] Stacy V., De Matteo R., Brew N., Sozo F., Probyn M.E., Harding R., Black M.J. (2009). The influence of naturally occurring differences in birthweight on ventricular cardiomyocyte number in sheep. Anat. Rec..

[35] Xue Q., Zhang L. (2009). Prenatal hypoxia causes a sex-dependent increase in heart susceptibility to ischemia and reperfusion injury in adult male offspring: Role of protein kinase C epsilon. J. Pharmacol. Exp. Ther..

[36] Netuka I., Szarszoi O., Maly J., Besik J., Neckar J., Kolar F., Ostadalova I., Pirk J., Ostadal B. (2006). Effect of perinatal hypoxia on cardiac tolerance to acute ischaemia in adult male and female rats. Clin. Exp. Pharmacol. Physiol..

[37] Patterson A.J., Chen M., Xue Q., Xiao D., Zhang L. (2010). Chronic prenatal hypoxia induces epigenetic programming of PKCε gene repression in rat hearts. Circ. Res..

[38] Bellio M.A., Pinto M.T., Florea V., Barrios P.A., Taylor C.N., Brown A.B., Lamondin C., Hare J.M., Schulman I.H., Rodrigues C.O. (2017). Hypoxic Stress Decreases c-Myc Protein Stability in Cardiac Progenitor Cells Inducing Quiescence and Compromising Their Proliferative and Vasculogenic Potential. Sci. Rep..

[39] Miao C.Y., Zuberbuhler J.S., Zuberbuhler J.R. (1988). Prevalence of congenital cardiac anomalies at high altitude. J. Am. Coll. Cardiol..

[40] Giordano F.J. (2005). Oxygen, oxidative stress, hypoxia, and heart failure. J. Clin. Investig..

[41] Williams S.J., Hemmings D.G., Mitchell J.M., McMillen I.C., Davidge S.T. (2005). Effects of maternal hypoxia or nutrient restriction during pregnancy on endothelial function in adult male rat offspring. J. Physiol..

[42] Ramirez-Bergeron D.L., Simon M.C. (2001). Hypoxia-inducible factor and the development of stem cells of the cardiovascular system. Stem Cells.

[43] Rios C., D’Ippolito G., Curtis K.M., Delcroix G.J., Gomez L.A., El Hokayem J., Rieger M., Parrondo R., de Las Pozas A., Perez-Stable C. (2016). Low Oxygen Modulates Multiple Signaling Pathways, Increasing Self-Renewal, While Decreasing Differentiation, Senescence, and Apoptosis in Stromal MIAMI Cells. Stem Cells Dev..

[44] Giussani D.A., Niu Y., Herrera E.A., Richter H.G., Camm E.J., Thakor A.S., Kane A.D., Hansell J.A., Brain K.L., Skeffington K.L. (2014). Heart disease link to fetal hypoxia and oxidative stress. Adv. Exp. Med. Biol..

[45] Hernandez I., Baio J.M., Tsay E., Martinez A.F., Fuentes T.I., Bailey L.L., Hasaniya N.W., Kearns-Jonker M. (2018). Short-term hypoxia improves early cardiac progenitor cell function in vitro. Am. J. Stem Cells.

[46] Ream M., Ray A.M., Chandra R., Chikaraishi D.M. (2008). Early fetal hypoxia leads to growth restriction and myocardial thinning. Am. J. Physiol. Regul. Integr. Comp. Physiol..

[47] Ostadal B., Ostadal P. (2014). Sex-based differences in cardiac ischaemic injury and protection: Therapeutic implications. Br. J. Pharmacol..

[48] Zhao X., Eghbali-Webb M. (2002). Gender-related differences in basal and hypoxia-induced activation of signal transduction pathways controlling cell cycle progression and apoptosis, in cardiac fibroblasts. Endocrine.

[49] Liu N., Bezprozvannaya S., Williams A.H., Qi X., Richardson J.A., Bassel-Duby R., Olson E.N. (2008). microRNA-133a regulates cardiomyocyte proliferation and suppresses smooth muscle gene expression in the heart. Genes Dev..

[50] Fasanaro P., D’Alessandra Y., Di Stefano V., Melchionna R., Romani S., Pompilio G., Capogrossi M.C., Martelli F. (2008). MicroRNA-210 modulates endothelial cell response to hypoxia and inhibits the receptor tyrosine kinase ligand Ephrin-A3. J. Biol. Chem..

[51] Cicchillitti L., Di Stefano V., Isaia E., Crimaldi L., Fasanaro P., Ambrosino V., Antonini A., Capogrossi M.C., Gaetano C., Piaggio G. (2012). Hypoxia-inducible factor 1-alpha induces miR-210 in normoxic differentiating myoblasts. J. Biol. Chem..

[52] Huang X., Ding L., Bennewith K.L., Tong R.T., Welford S.M., Ang K.K., Story M., Le Q.T., Giaccia A.J. (2009). Hypoxia-inducible mir-210 regulates normoxic gene expression involved in tumor initiation. Mol. Cell..

[53] Mutharasan R.K., Nagpal V., Ichikawa Y., Ardehali H. (2011). microRNA-210 is upregulated in hypoxic cardiomyocytes through Akt- and p53-dependent pathways and exerts cytoprotective effects. Am. J. Physiol. Heart Circ. Physiol..

[54] Lv J.X., Zhou J., Tong R.Q., Wang B., Chen X.L., Zhuang Y.Y., Xia F., Wei X.D. (2019). Hypoxia-induced miR210 contributes to apoptosis of mouse spermatocyte GC2 cells by targeting Kruppellike factor 7. Mol. Med. Rep..

[55] Endo K., Naito Y., Ji X., Nakanishi M., Noguchi T., Goto Y., Nonogi H., Ma X., Weng H., Hirokawa G. (2013). MicroRNA 210 as a biomarker for congestive heart failure. Biol. Pharm. Bull..

